# Through‐Bond Energy Transfer Cassette with Dual‐Stokes Shifts for “Double Checked” Cell Imaging

**DOI:** 10.1002/advs.201700229

**Published:** 2017-10-27

**Authors:** Xiangdong Xue, Shubin Jin, Zhipeng Li, Chunqiu Zhang, Weisheng Guo, Liming Hu, Paul C. Wang, Jinchao Zhang, Xing‐Jie Liang

**Affiliations:** ^1^ Chinese Academy of Sciences (CAS) Center for Excellence in Nanoscience and CAS Key Laboratory for Biological Effects of Nanomaterials and Nanosafety National Center for Nanoscience and Technology No. 11 Beiyitiao Zhongguancun Beijing 100190 China; ^2^ University of Chinese Academy of Sciences Beijing 100049 China; ^3^ Beijing Municipal Institute of Labor Protection Beijing 100054 China; ^4^ School of Life Sciences Beijing University of Technology Beijing 100124 China; ^5^ College of Science and Engineering Fu Jen Catholic University Taipei 24205 Taiwan; ^6^ Laboratory of Molecular Imaging Department of Radiology Howard University Washington DC 20060 USA; ^7^ College of Chemistry and Environmental Science Hebei University Baoding 071002 China

**Keywords:** cell imaging, energy transfer, fluorescence, pseudo‐Stokes shift

## Abstract

Organic dyes generally suffer from small Stokes shift that usually leads to self‐quenching and ‐gaining errors during the fluorescent imaging process. Here, a through‐bond energy transfer (TBET) cassette is developed with large Stokes shift to pursue precise cell imaging. The TBET is constructed by covalently conjugated tetraphenylethene (acts as donor) and rhodamine (acceptor) through an acetylene bond. The constructed TBET cassette distinctly behaves as dual‐Stokes shifts, including a large pseudo‐Stokes shift caused by energy transfer, from donor's absorption to acceptor's emission (up to 260 nm) and a smaller Stokes shift of acceptor molecules itself. Due to the intrinsic dual‐Stokes shifts, TBET cassette exhibits specific “dual distinct absorbances, single shared emission” properties, which can be excitated under two different laser channels. By colocalization of the imaging readouts of these two channels, the precisely “double checked” fluorescent imaging is achieved in living cells.

Fluorescent imaging techniques have been broadly applied for biological and medical research, due to their high sensitivity, superior biocompatibility, and great versatilities for cell investigation, but minimally disturbing cells proliferations.^1^ Small‐molecule organic dyes with high quantum yield, such as rhodamine (Rho),^2^ fluorescein,^3^ boron‐dipyrromethene (BODIPY),^4^ and cyanine derivatives,^5^ are extensively served as contrast agents for fluorescent imaging. However, these dyes generally behave as a small Stokes shift, which leads to fluorescence self‐quenching and gaining errors due to crosstalk of the very adjacent excitation and emission.^6^ To address this problem, organic dyes with large Stokes shift need to be developed.

Since it is still challenging to develop single organic dyes with intrinsic large Stokes shift, scientists tend to seek multichromophore with donor–acceptor architectures as alternatives. Based on the principle of energy transfer, fluorescence resonance energy transfer (FRET) has been widely utilized to obtain large pseudo‐Stokes shift.^7^ FRET undergoes through space energy transfer pathway that suffers from low energy transfer efficiency (ETE%),^8^ even the large pseudo‐Stokes shift of FRET can be easily realized, the fluorescence generated by energy transfer is usually feebly performed. Furthermore, FRET occurrence requires specific spectra overlap between the emission of donor and absorption of acceptor, which largely constrains the design of desirable FRET dyads. The construction of through‐bond energy transfer (TBET) exhibits more flexibilities.^9^ TBET dyads are not mandatory to meet requirements of spectral overlap, and can be constructed by any kinds of fluorescent molecules, theoretically. Meanwhile, the excitation energy can be directly transferred to acceptor molecules through chemical bonds with extremely high ETE%.^10^ Hence, TBET dyads not only perform large pseudo‐Stokes shift, but also undergo highly efficient energy transfer for fluorescent signal gains. The design of TBET molecules was more accessible and flexible for the development of fluorescent dyes with large Stokes‐shift.

For biological fluorescent imaging, undesired spontaneous fluorescence inevitably accompanies with targeted fluorescent signal, such as autofluorescence.^11^ Undesirable fluorescence usually confounds targeted fluorescence and confuses the real imaging results. If a single fluorescent molecule can be excitated by multiple lasers, and the fluorescence readouts are able to be compared and screened, the undesirable fluorescence may be sorted out from the targeted signals.

To realize more specific biological imaging, fluorescent molecules are supposed to be with large Stokes shift to deplete spectra crosstalk caused imaging interference; moreover, they are requested to possess multiple absorbance wavelengths, which can realize multichannel imaging, so that the fluorescence readouts can be screened. Hence, we constructed a TEBT cassette, which not only behaved as two distinct absorbances, that is, the absorbances of donor and acceptor respectively, but also was born with large pseudo‐Stokes shift and perfectly meets the requirements for specific biological fluorescent imaging.

In TBET cassette, molecular structures of donor and acceptor cannot be planar, in case the newly constructed fluorescence system behaves as a single conjugated dye.[[qv: 10a]] Hence, a typical aggregation‐induced emission (AIE) molecule, tetraphenylethylene (TPE), was served as energy transfer donor, due to the nonplanar molecular structures of AIE molecules.^12^ Not only that, TPE molecules emit no fluorescence in dissolved state due to fast nonradiative decay, so that the fluorescence leakage can be largely mitigated in some cases that TBET efficiency is not high enough.^13^ TPE molecules are easy to be modified and usually behave as relative large Stokes shifts (typically 150–200 nm) and broader emission band,^14^ and thus beneficial to the construction of TBET with ultralarge pseudo‐Stokes shifts and high ETE%. Then, an Rho derivative was conjugated to TPE as acceptor, because of the large molar absorptivity and high quantum yield.^15^ The excellent photophysical properties of Rho are beneficial for the improvement of the signal sensitivity, easpecially for biological imaging, as trace amount of Rho molecules can gain strong signal readouts, which may suppress the autofluorescence comes from biology systems. The chemical synthesis of the TBET cassette was shown in **Figure** **1**. To realize energy transfer process, appropriate connection between the donor and acceptor should be energy transferable. As designed, TPE and Rho were covalently conjugated through a most commonly used energy transfer chemical bond (acetylene bond)^13^ and formed TPE–Rho conjugate (TRc). Based on nuclear magnetic resonance (NMR) and high‐resolution mass spectrometry (HRMS) characteristics, TBET cassette (TRc) was successfully constructed (Figures S1–S4, Supporting Information).

**Figure 1:**
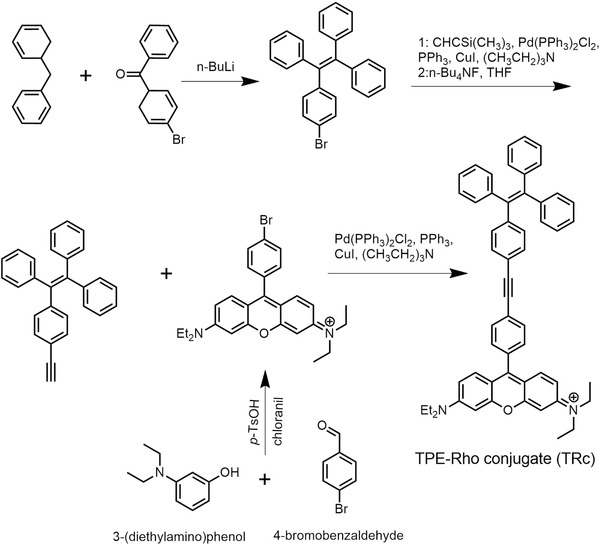
Chemical synthesis of the TBET cassette.

TPE was typical AIEgen and did not show fluorescence in the dissolved state, due to the nonradiative decay. To make the energy transfer easily to be observed, we checked the AIE behaviors of TPE and tried to testify the energy‐transfer related optical behaviors of TRc when the donor was luminant. TPE molecules (10 × 10^−6^ m) were dissolved in DMSO/water mixtures with different fractions of water (*f*_w_%) for AIE investigation. As shown in Figure S5 in the Supporting Information, TPE molecules showed less fluorescence when they were well dissolved in dimethyl sulfoxide (DMSO), and the fluorescence became distinguishable when the water fractions were ≥60%. Hence, the DMSO/H_2_O (40/60, v/v%) mixture was employed as solvent for the optical measurements. TRc molecules were supposed to behave two distinct absorptions, which were originated from donor and acceptor, respectively. As shown in **Figure** **2**a, TPE molecules showed obvious single ultraviolet (UV) absorption at 315 nm, and Rho exhibited strong absorption at 570 nm. When they were conjugated together, TRc showed absorptions of both TPE (315 nm) and Rho (570 nm). We reasoned that the fluorescence of TRc can be excitated by either donor's or acceptor's absorption.

**Figure 2:**
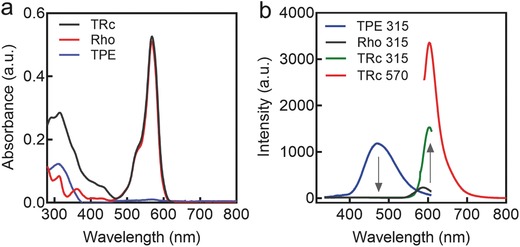
UV–vis and fluorescence behaviors of the TBET cassette. a) UV–vis spectra of the donor (TPE), acceptor (Rho) and TBET cassette (TRc); b) The fluorescence behaviors of TRc, and its precursors (TPE and Rho). They were excitated by two distinct absorbance wavelengths: 315 and 570 nm. 10 × 10^−6^ m of TPE, Rho, and TRc were dissolved in DMSO/H_2_O (40/60, v/v%) for the optical measurements.

To manifest this hypothesis, fluorescence behaviors of TRc molecules were investigated. In the fluorescence spectra (Figure 2b), TPE molecules exhibited strong fluorescence intensity at 460 nm when they were excitated at 315 nm (maximum absorbance of TPE showed in Figure 2a). Rho molecules only showed neglectable emission at ≈590 nm when excitated at 315 nm (the optimal absorbance of TPE). In comparison, TRc molecules exhibited strong fluorescence at ≈600 nm when they were excitated by the excitation of donor molecule (TPE) at 315 nm, but no distinguishable fluorescence at ≈460 nm. In comparison, TRc molecules exhibited much stronger fluorescence at 590–600 nm than Rho when they were excitated under the same wavelength (315 nm) and showed no emission at 460 nm (the maximum emission of TPE), indicating that highly efficient energy transfer happened between the TPE (donor) and Rho (acceptor) in TRc molecules. In the energy transfer system, fluorescence disappearance of TPE was ascribed to high efficient TBET, and the extensive enhancement on Rho's emission was caused by the emissive energy that was transferred from TPE. Meanwhile, TRc can also be excitated at 570 nm and showed the higher emission intensity than that excitated at 315 nm (energy transfer emission peak), as 570 nm is the maximum excitation of Rho molecules. The disappearance of donor's emission implied that the ETE% was highly efficient. We then calculated the ETE%,[[qv: 13a]] as shown in Figure S6 in the Supporting Information, the ETE% of our TBET cassette reached 99.9%, exhibiting extremely high energy transfer capacity. According to the results of UV–vis absorptions and fluorescence behaviors, our TBET cassette behaves as two distinct absorbances. As such, TBET cassette owns dual‐Stokes shifts, including a large pseudo‐Stokes shift caused by energy transfer, from donor's absorption to acceptor's emission (up to ≈260 nm) and a smaller Stokes shift of acceptor molecules (Rho) itself, from 570 to 590 nm (≈20 nm), in which the large pseudo‐Stokes shift can mitigate the “crosstalk” imaging interference that may occur during single Rho‐dominated imaging process. TRc can also be excitated by two distinct lasers and emitted the excitation energy at same emission band, indicating that our TBET cassette possesses “dual distinct absorbances, single shared emission” photo‐physical properties. Hence, TBET cassette meets the criterion to realize “double checked” cell imaging. As illustrated in **Scheme** **1**, the dual‐absorbances allow the fluorescence of TRc can be excitated and captured in two distinct channels, the fluorescence signals were obtained at the same wavelength, which generated from the very same chromophore (Rho). By colocalization and comparison of the fluorescence readouts from two channels, undesirable fluorescence can be directly sorted out. The fluorescence that is not colocalized was considered as undesirable fluorescence, as the fluorescent signals of the TBET cassette were originated from two distinct absorbances, but emitted from the same chromophore (the acceptor).

**Scheme 1:**
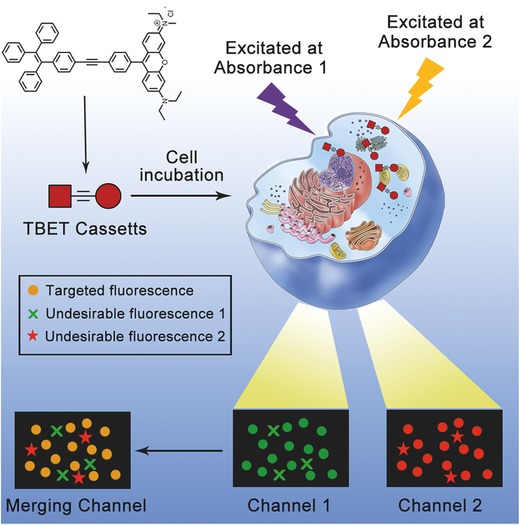
Schematic illustration of the “double checked” cell imaging of TRc by means of through‐bond energy transfer (TBET).

TBET cassette was implemented for “double checked” imaging in living cells. We first incubated TRc with cells for biocompatibility evaluation. As shown in **Figure** **3**, TRc exhibited no significant cytotoxicity from 0.1 × 10^−6^ to 10 × 10^−6^ m, indicating that our TBET cassette performed good biocompatibility and was suitable for biological applications.

**Figure 3:**
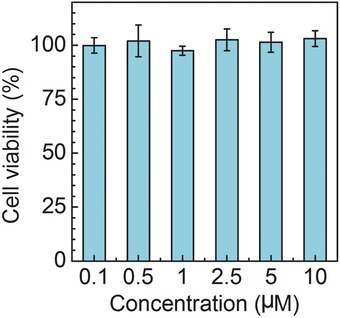
Cell viabilities of TRc‐treated MCF‐7 breast cancer cells for 24 h.

To evaluate the fluorescence behaviors of the TBET cassette at cellular level, 2.5 × 10^−6^ m of TRc were incubated with MCF‐7 breast cancer cells for 30 min. As control, same molar of TPE (donor) and Rho (acceptor) was also incubated with cells under identical procedures. To investigate the subcellular distributions of these different fluorescent molecules, MCF‐7 cells were observed by confocal laser scanning microscopy (CLSM) under three different laser channels, which corresponded to donor channel (TPE, Ex/Em, 405/460 nm), acceptor channel (Rho, Ex/Em, 543/590 nm), and energy transfer channel (TRc, Ex/Em, 405/590 nm). In TPE treated group (top panel of **Figure** **4**), barely detectable fluorescence signal was observed either in acceptor channel (Rho 543/590) or energy transfer channel (TBET 405/590), whereas blue fluorescence of TPE was only observed in donor channel (TPE 405/460). The subcellular distributions of the blue fluorescence indicated that donor molecules (TPE) were mainly dispersed in cytoplasm, and showed no evidence of nucleic distribution. Similarly, fluorescence of Rho in acceptor channel (Rho 543/590) was clearly observed in Rho treated cells (middle panel of Figure 4), and uniformly distributed in the cytoplasm, no detectable fluorescence appeared in the other channels. These results suggested that single fluorescence of donor or acceptor can only be observed through their own fluorescence channels, but not be suitable for comparatively, multi‐channel cell imaging. We then observed the subcellular distributions of TRc molecules through three channels (bottom panel of Figure 4). In donor channel (TPE 405/460), no distinguishable fluorescence signal was observed, as TBET was so effective that almost all emissive energy of donors transferred to the acceptors, showing consistent behaviors with the fluorescence spectra (Figure 2b). In consequence, strong fluorescence in energy transfer channel (TBET 405/590) was observed, which was excitated by donor's excitation, but harvested at acceptor's emission. At the same time, in acceptor's channel (Rho 543/590), the fluorescence showed the same subcellular patterns with that in energy transfer channel, which was in line with the fluorescence spectra results showed in Figure 2b. Since the signal readouts of TBET 405/590 and Rho 543/590 were excitated from two distinct absorbances of donor and acceptor, and consequentially emitted fluorescence from a single shared chromophore (Rho), the signal readouts in these two channels were supposed to perform large colocalization areas. Therefore, the colocalization efficiency was denoted in forms of Pearson's correlation coefficient.^16^ The colocalization efficiency between TBET 405/590 and Rho 543/590 channels in TRc treated cells is shown in **Figure** **5**a, the Pearson's correlation coefficient was around 0.9, indicating very high colocalization efficiency. The TPE and Rho treated groups were employed as control, in which the TPE 405/460 and Rho 543/590 channels showed extremely low colocalization with their energy transfer channel (TBET 405/590), indicating single dye cannot realize “double checked” cell imaging. In statistics, the colocalization efficiency of TRc molecules showed strongly significant differences (*p* < 0.0001) by compared with their precursors (TPE and Rho). Meanwhile, we found that TRc exhibited different subcellular behaviors, comparing to its donor and acceptor molecules: they preferentially accumulated in a particular region. We then costained the cells with a commercial lysotracker (LysoTracker Deep Red) and found that the fluorescence from dual channels of TRc was showing excellent colocalization with commercial lysotracker, indicating that our TRc molecules were potentially capable of lysosomes tracking. On the contrary, neither TPE nor Rho exhibited better lysosomes colocalization pattern. The colocalization efficiency of TPE, Rho, and TRc treated cells were evaluated in Figure 5b. In TRc treated cells, both 543/590 and 405/590 channels showed high colocalization efficiency with lysosomes, the Pearson's correlation coefficient that is calculated by compared 543/590 channel to lysotracker channel was up to 0.8, and 405/590 channel was around 0.7, indicating large cololization areas. In statistics, both imaging channels of 543/590 and 405/590 showed significant differences to TPE and Rho. The reasons why TRc preferencially stained the lysosomes may be ascribe to two aspects, i) TRc molecules self‐assembled into nanoparticles (Figure S7, Supporting Information). Since the nanoparticles were generally ingested into cells through endocytosis and have large chances to be transported to lysosomes.^17^ TRc molecules can ignite lysosomes when they were released from TRc nanoaggregation; ii) By comparing the chemical structures of the TRc with some commercially available lysotrackers from ThermoFisher Scientific (Figure S8, Supporting Information), and publishing lysosome probes.^18^ The weakly basic amines groups on TRc help to selectively accumulate in cellular compartments with low internal pH and can be used to investigate the biosynthesis and pathogenesis of lysosomes.^19^ Furthermore, TRc showed advantages comparing with commercial lysotrackers: they can be excitated with two distinct absorbances and emitted fluorescence from the same chromophore, showed unique characterization of “dual distinct absorbances, single shared emission,” and thus exhibited advanced “double checked” cell imaging feature.

**Figure 4:**
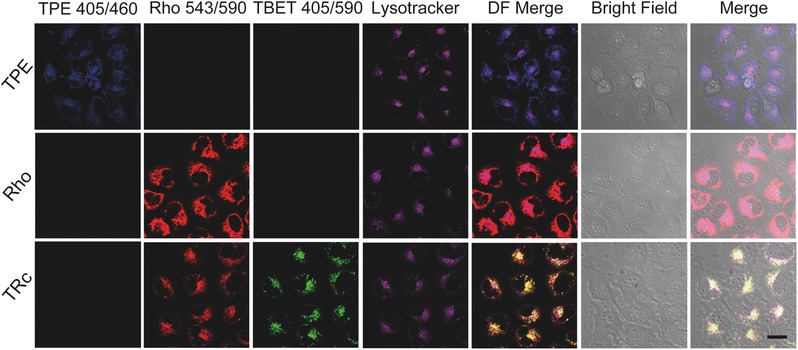
CLSM images of donor (TPE), acceptor (Rho), and TBET cassette (TRc), incubating with MCF‐7 cancer cell for 30 min, respectively. The concentrations of all materials were set to 2.5 × 10^−6^ m. Channel TPE 405/460 means excitated by 405 nm and received the fluorescence signal in 460 ± 10 nm; Rho 543/590, excitated at 543 nm, received at 590 ± 10 nm; TBET 405/590, excitated at 405 nm, received at 590 ± 10 nm. The lysosomes were stained with commercial lysotracker, LysoTracker Deep Red, excitated at 630 nm, and received at 670 ± 10 nm. The scale bar is 20 µm.

**Figure 5:**
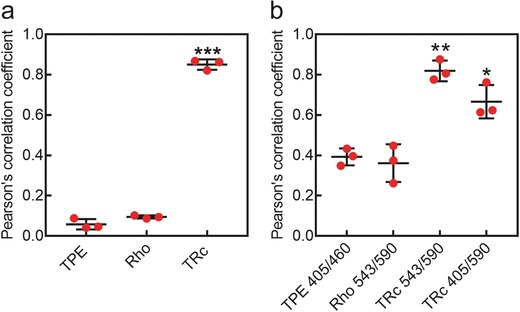
Quantification of colocalization. a) Colocalization efficiency between the fluorescent channels of TPE (405/460), Rho (543/590), and TRc (543/590) treated groups and the energy transfer channels (405/590). ***, *p* < 0.0001. b) Colocalization efficiency between the TPE, Rho, and TRc treated groups and their corresponding lysotracker channel. *, *p* < 0.05; **, *p* < 0.01. The Pearson's correlation coefficient was employed to quantitate the colocalization efficiency. Three repeats of each treatment were processed with Imagej to obtain Pearson's correlation coefficient.

TRc‐stained cells were then scanned in three‐dimensional (3D) mode for pinpointing the undesirable fluorescence during the imaging process. The results were shown in **Figure** **6**, TRc was excitated by two distinct absorbances, and dual‐channel fluorescence was collected and compared through colocalization. We focused on a specific region of interest and amplified them in the *X*, *Y*, and *Z* axes, respectively (shown as amplified 1, 2, and 3 in Figure 6). We found that some fluorescence region did show non‐colocalization. In amplified 1, green arrows marked the fluorescence only showed up in 405/590 channel, and non‐colocalized with the fluorescence excitated by Rho's excitation, denoting undesirable signal generated under the excitation of 405 nm. Meanwhile, some “red” unmerged fluorescence was observed as well (pointed out by red arrow), the non‐colocalized fluorescence was considered as suspected undesirable fluorescence generated by the process of CLSM observation under 543 nm lasers. On the contrary, the colocalized fluorescent areas (yellow fluorescence) were considered as the real and “double checked” subcellular distributions of TRc. These results suggested that TBET cassette can precisely, “double checked” stain the cells. By virtue of the “dual distinct absorbances, single shared emission” behaviors of the TBET cassette, TRc was capable of visualizing the cells by fluorescence with minimized imaging interferences.

**Figure 6:**
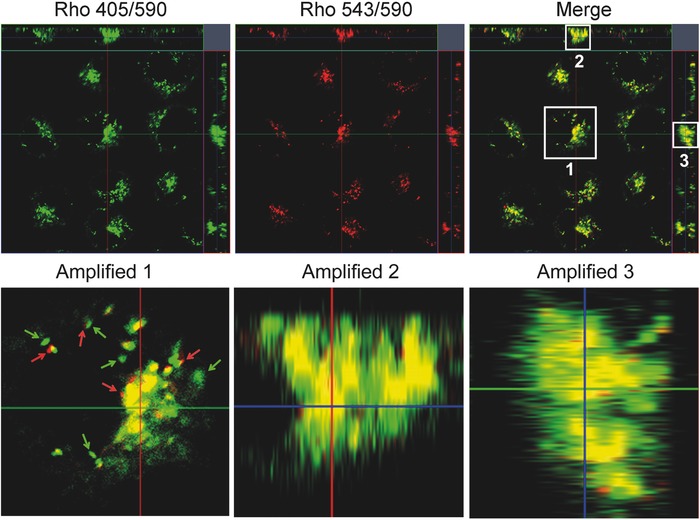
3D‐simulated CLSM images of TRc, incubating with MCF‐7 cancer cell for 30 min. The red arrow denoted the “nondouble checked” red fluorescence; and the green arrow indicated the “nondouble checked” green fluorescence.

In this study, we developed a novel through‐bond energy transfer fluorescent dye with unique “dual distinct absorbances, single shared emission” characteristics for “double checked,” precise cell imaging. In TBET cassette, fluorescence can be excitated either at the range of donor's absorbance or acceptor's absorbance and thus giving two distinct fluorescent readouts. By colocalizing of two groups of fluorescence signals, undesirable fluorescence that always misleads the imaging results can be sorted out. After colocalization, the fluorescence does not merge with their counterparts is considered as undesirable fluorescence emitted from cells or during the CLSM observation process. The fluorescence signals that can be perfectly merged were considered as the real subcellular positions of the fluorescent dye. The “double checked” TBET cassette provides a straightforward solution for depletion of undesirable fluorescence which may confound fluorescent imaging in vitro. Our strategy smartly achieves precise cell imaging only based on fluorescence dye and does not need to spend huge expenses on tackling undesirable fluorescence disturbance by improving the instruments and thus offering great promise for inspiring scientists to develop more sophisticated and specific imaging materials.

## Experimental Section

*Chemicals and Instruments*: All chemicals and regents were purchased and used as received without further purification. ^1^H nuclear magnetic resonance (NMR) spectrum was measured on a Bruker AV 300 spectrometer in chloroform with tetramethylsilane (δ = 0) as an internal reference. Mass spectrum was taken on a high‐resolution mass spectrometry (HRMS, Finnigan Surveyor MSQ‐plus, Thermo Inc.). UV absorbance was evaluated by a UV–vis spectrometer (Lambda 950, Perkin Elmer Inc.); fluorescence behaviors of the materials were tested by a fluorescence spectrometer (LS55, Perkin Elmer Inc.) and cellular fluorescence distributions were observed by confocal laser scanning microscope (CLSM, LSM710, Carl Zeiss).

*Synthesis of TRc*: TPE and Rho were prepared following the literature method.^14^ A mixture of TPE (120.0 mg, 0.25 mmol), Rho (160.0 mg, 0.25 mmol), copper(I) iodide (4.0 mg, 0.015 mmol), Pd(PPH_3_)_2_Cl_2_ (11.5 mg, 0.015 mmol), and PPh_3_ (4.5 mg, 0.015 mmol) was dissolved in a solvent mixture of dry triethylamine (3 mL) and anhydrous tetrahydrofuran (THF) (3 mL) under nitrogen (N_2_ ) protection. The mixture was stirred at 70 °C for 24 h. After removal of solvent under vacuum, the residue was purified by column chromatography (silica gel; 0–10%, MeOH/DCM, linear gradient) to give a purple solid; yield 26.0 mg (10%). ^1^H NMR (300 MHz, CDCl_3_) δ8.79 (s, 1H), 8.39 (s, 2H), 7.76 (d, *J* = 8.1Hz, 2H), 7.42 (s,1H), 7.4 (d, *J* = 3.1Hz, 2H), 7.37 (s, 2H), 7.35 (s,1H), 7.19–7.13 (m, 9H), 7.12–7.04 (m, 8H), 6.98 (s, 2H), 6.95 (s, 1H), 6.92 (s, 1H), 3.7 (q, *J* = 6.9Hz, 8H), 1.38 (t, *J* = 7.0 Hz, 12H). HRMS: *m/z* 753.3835 (M^+^, calculated 753.3839).

*Transmission electron microscopy (TEM) Observation of TRc Nanoaggregates*: 1 µL TRc DMSO solution (100 × 10^−3^ m) was dropped into 999 µL Milli Q water, with sonication for 30 s, the 100 × 10^−6^ m TRc nanoaggregates were obtained. The TRc nanoaggregates were then diluted to 10 × 10^−6^ m and dropped on copper grid for TEM observation after naturally dried.

*Calculation of the Energy Transfer Efficiency (ETE%)*: The ETE% was calculated by the equation: ETE% = [1 − (*I*_DA_/*I*_D_)] × 100. *I*_DA_ denotes the integral of the emission spectra of the donor in the presence of an acceptor; *I*_D_ is the integral of the emission spectra of donor molecules without an acceptor presence.

*Cell Viabilities Evaluation*: MCF‐7 cells were seeded at 5 × 10^3^ cells well^−1^ in a 96‐well plate, preincubated for 24 h, then incubated with TRc for 24 h with concentrations ranging from 0.1 × 10^−6^ to 10 × 10^−6^ m. The medium was then replaced with 100 µL fresh medium containing 0.5 mg mL^−1^ MTT and after 2 h, the MTT solution was replaced with 150 µL DMSO solution. The absorbance was measured at 570 nm with a reference wavelength of 630 nm using an Infinite M200 microplate reader (Tecan, Durham, USA). Untreated cells in medium were used as controls.

*Fluorescence Distributions of TPE, Rho and TRc in Living Cells*: 10^5^ MCF‐7 cells were seeded into 35 mm glass bottom dishes, incubated at 37 °C for 24 h until completely being attached, then incubated with 2.5 × 10^−6^ m TPE, Rho, and TRc at 37 °C for 30 min, respectively. Cells were then rinsed with phosphate‐buffered saline (PBS) twice and incubated with LysoTracker Deep Red (1000 times dilution in PBS) for lysosomes staining. After 10 min incubation, cells were rinsed with PBS twice and imaged using a CLSM (LSM 710, Carl Zeiss) with excitation at 405 nm for TPE, 543 nm for Rho, both 405 and 543 nm for TRc, and 630 nm for LysoTracker Deep Red.

## Conflict of Interest

The authors declare no conflict of interest.

## Supporting information

Supplementary
