# Supplementary material for: Through‐Bond Energy Transfer Cassette with Dual‐Stokes Shifts for “Double Checked” Cell Imaging
Source: Adv Sci (Weinh). 2017 Oct 27;4(12):1700229. doi: 10.1002/advs.201700229 (PMC5737226; doi:10.1002/advs.201700229)
Supplement: Supplementary file 1 — Supplementary [file ADVS-4-na-s001.pdf]

Copyright WILEY-VCH Verlag GmbH & Co. KGaA, 69469 Weinheim, Germany, 2017.

ADVANCED SCIENCE
Open Access

Supporting Information

for *Adv. Sci.,* DOI: 10.1002/advs.201700229

Through-Bond Energy Transfer Cassette with Dual-Stokes Shifts for “Double Checked” Cell Imaging

*Xiangdong Xue, Shubin Jin, Zhipeng Li, Chunqiu Zhang, Weisheng Guo, Liming Hu, Paul C. Wang, Jinchao Zhang, and Xing-Jie Liang**

Supporting Information

Through-Bond Energy Transfer Cassette with Dual-Stokes Shifts for "Double Checked" Cell Imaging

Xiangdong Xue,[†,a,c] *Shubin Jin,*[†, a,b,c] *Zhipeng Li,*[d] *Chunqiu Zhang,*[a] *Weisheng Guo,* [a, c] *Liming Hu,*[d] *Paul C Wang,* [e,f] *Jinchao Zhang,* [g] *Xing-Jie Liang**[,a, c]

[a] Chinese Academy of Sciences (CAS) Center for Excellence in Nanoscience and CAS Key Laboratory for Biological Effects of Nanomaterials & Nanosafety, National Center for Nanoscience and Technology, No. 11 Beiyitiao, Zhongguancun, Beijing 100190, China.
E-mail: liangxj@nanoctr.cn
[b] Beijing Municipal Institute of Labor Protection, Beijing, 100054, China.
[c] University of Chinese Academy of Sciences, Beijing, China 100049.
[d] Beijing University of Technology, Beijing, 100124, China.
[e] Fu Jen Catholic University, Taipei, Taiwan 24205.
[f] Laboratory of Molecular Imaging, Department of Radiology, Howard University, Washington, D.C. USA 20060.
[g] College of Chemistry & Environmental Science, Hebei University, Baoding 071002, China
[†] These authors contributed equally.

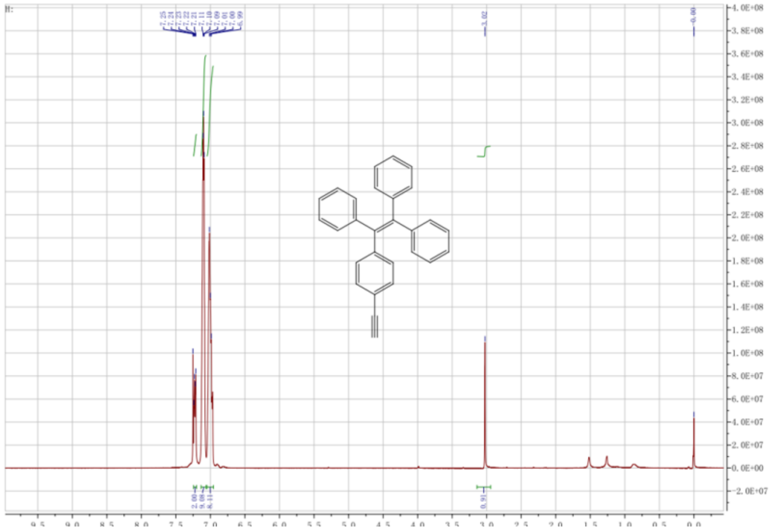

Figure S1 ^{1}H-NMR spectrum of TPE.

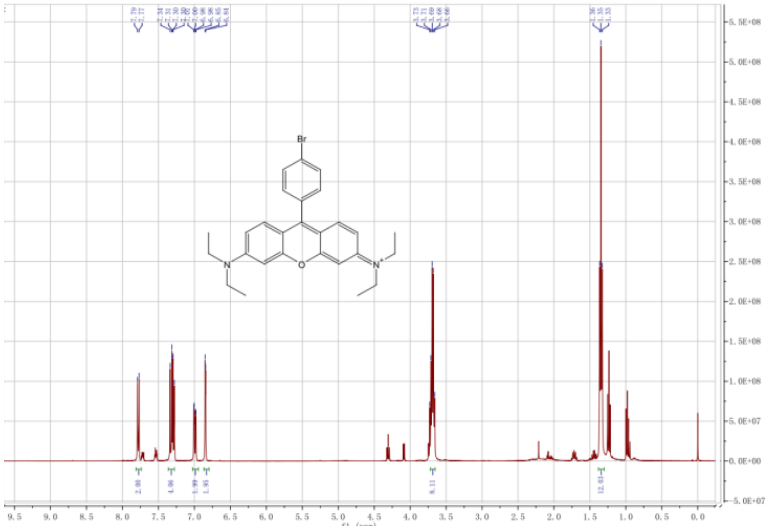

Figure S2 ^{1}H-NMR spectrum of Rho.

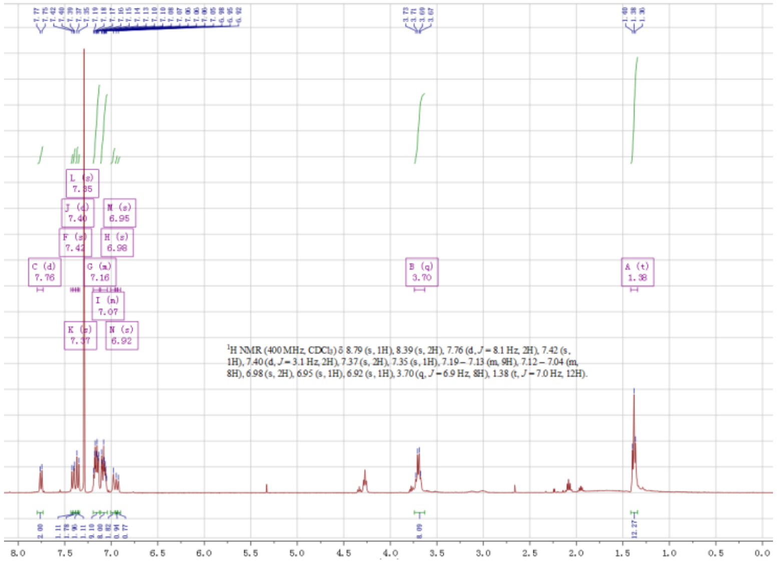

Figure S3 ^{1}H-NMR spectrum of TRc

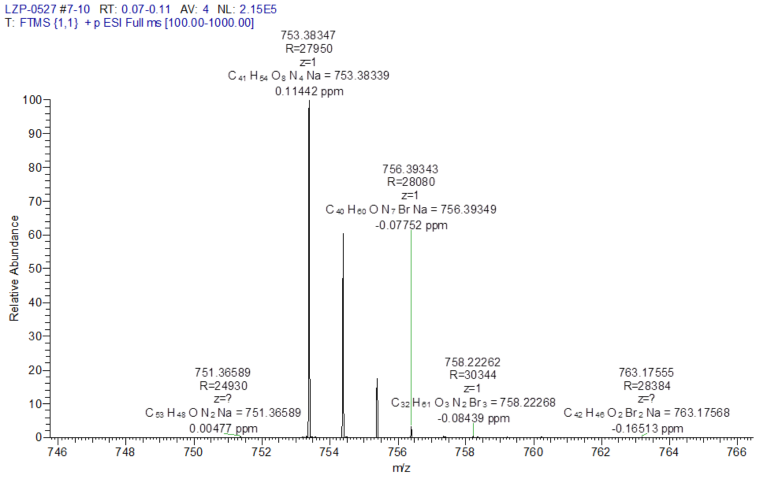

Figure S4 HR-MS of TRc.

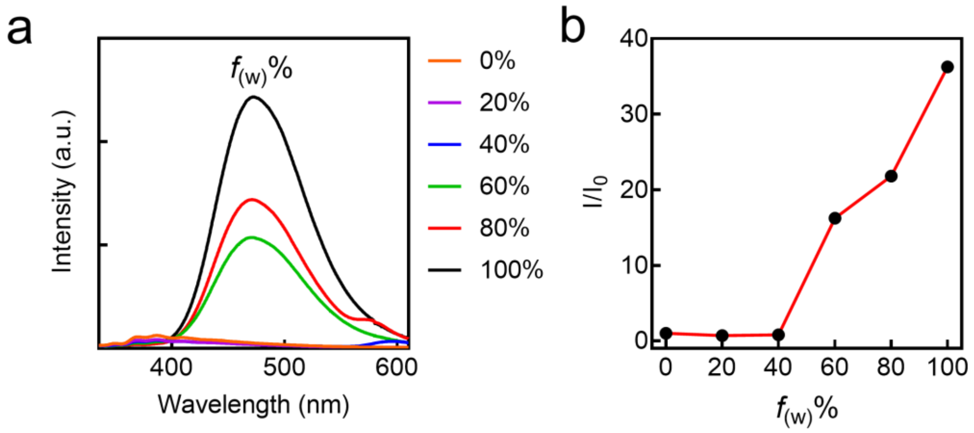

Figure S5. AIE behaviors of TPE. a) FL spectra of TPE (10 μM) in DMSO/water mixtures with different fractions of water, $f_{(w)}$%. b) Plot of FL intensity (I) of TPE at 460 nm versus DMSO/water mixtures with different $f_{(w)}$%. I_0 is the FL intensity of TPE in pure DMSO solution; excitation wavelength is 315 nm. DMSO is good solvent of TPE, and makes it well dissolved. Water is poor solvent of TPE, which makes TPE aggregated, and thus performed AIE behaviors.

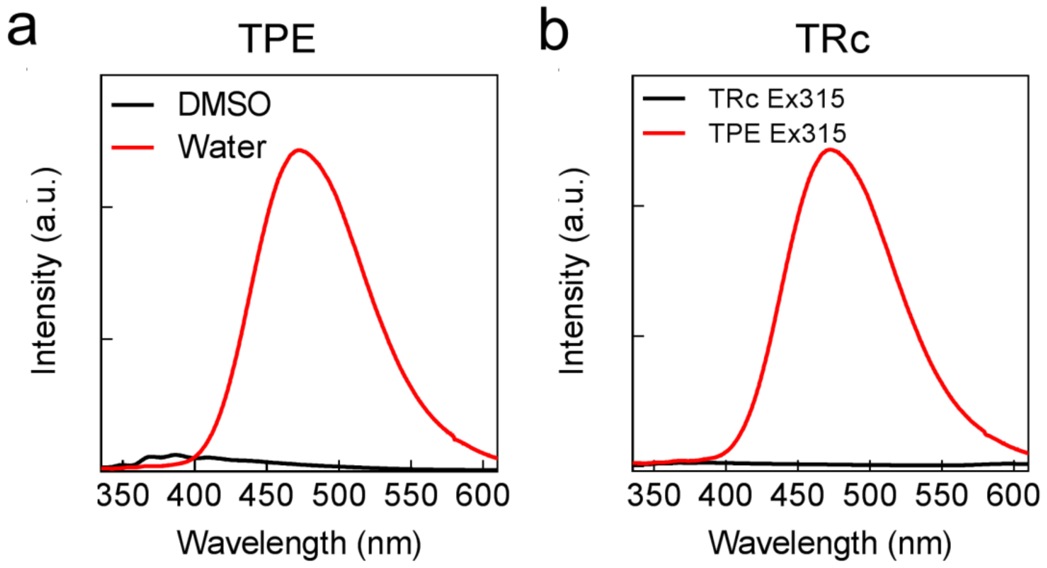

Figure S6. The fluorescence behaviors of TPE and TRc (ETE% calculation). a) The fluorescence of TPE in good (DMSO) and poor solvent (Water). In pure water, TPE performed highest AIE fluorescence (~36 times higher than that dissolved in good solvent, Figure S5). b) The fluorescence behaviors of same molar (10 μM) of TPE and TRc in water by excitated with donor's excitation (315 nm). Since the TPE performed AIE fluorescence in water, we calculated the energy transfer efficiency of TRc in water solution. In poor solvent (water), TRc followed an energy transfer relay, and emitted no fluorescence, i.e. TPE transferred its emission to the acceptor, due to the high energy transfer efficiency, and Rho quenched both fluorescence of TPE and Rho itself by ACQ, which caused by "π-π" stacking between the Rho molecules. Therefore, the fluorescence of TRc in water showed no fluorescence both at donor and acceptor's emissions band. The energy transfer efficiency (ETE%) was calculated by the equation: ETE%=[1-(I_{DA}/I_D)]*100. I_{DA} denotes the integral of the emission spectra of the donor in the presence of an acceptor; I_D is the integral of the emission spectra of donor molecules without an acceptor presence. The ETE% was 99.9% by calculation.

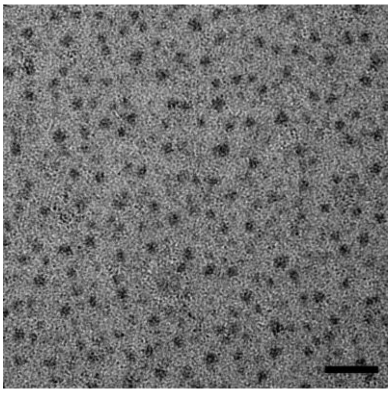

Figure S7 TEM image of TRc nano-aggregates. The scale bar is 100 nm.

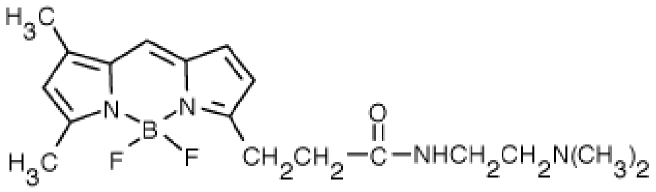

LysoTracker™ Green DND-26

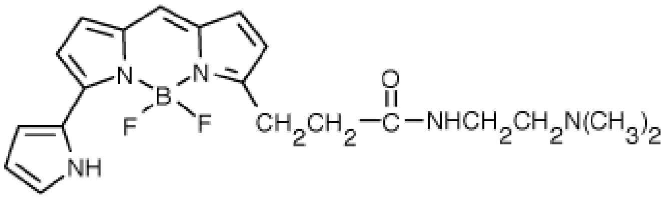

LysoTracker™ Red DND-99

Figure S8 Chemical structures of commercial available lysotrackers.
